# An autonomous cycle of data analysis tasks for the clinical management of dengue

**DOI:** 10.1016/j.heliyon.2022.e10846

**Published:** 2022-09-29

**Authors:** William Hoyos, Jose Aguilar, Mauricio Toro

**Affiliations:** a Grupo de Investigaciones Microbiológicas y Biomédicas de Córdoba, Universidad de Córdoba, Montería, Colombia; b Grupo de Investigación en I+D+i en TIC, Universidad EAFIT, Medellín, Colombia; c Centro de Estudios en Microelectrónica y Sistemas Distribuidos, Universidad de Los Andes, Mérida, Venezuela; d Universidad de Alcalá, Departamento de Automática, Alcalá de Henares, Spain

**Keywords:** Dengue, Autonomic computing, Clinical decision-support system, Computational intelligence, Data analysis

## Abstract

Dengue is the most widespread vector-borne disease worldwide. Timely diagnosis and treatment of dengue is the main objective of medical professionals to decrease mortality rates. In this paper, we propose an autonomous cycle that integrates data analysis tasks to support decision-making in the clinical management of dengue. Particularly, the autonomous cycle supports dengue diagnosis and treatment. The proposed system was built using machine learning techniques for classification tasks (artificial neural networks and support vector machines) and evolutionary techniques (a genetic algorithm) for prescription tasks (treatment). The system was quantitatively evaluated using dengue-patient datasets reported by healthcare institutions. Our system was compared with previous works using qualitative criteria. The proposed system has the ability to classify a patient's clinical picture and recommend the best treatment option. In particular, the classification of dengue was done with 98% accuracy and a genetic algorithm recommends treatment options for particular patients. Finally, our system is flexible and easily adaptable, which will allow the addition of new tasks for dengue analysis.

## 1 Introduction

Dengue is an arthropod-borne viral disease transmitted by *Aedes* mosquitoes, mainly *Aedes aegypti* and *Aedes albopictus* [1]. Currently, this infection is considered the most important arbovirosis worldwide in terms of morbidity, mortality and economic impact [2]. Between epidemiological weeks 1 and 49 of 2021, 1,173,674 dengue cases in the Americas region were reported, with a cumulative incidence rate of 118 cases per 100,000 inhabitants. In this period, the most affected subregions were the Southern Cone with a cumulative incidence of 323 cases/100,000 inhabitants, and the Andean subregion with 89 cases/100,000 inhabitants. Within the Andean subregion, Colombia is in third place with an incidence of 95 cases per 100,000 inhabitants, surpassed by Peru and Ecuador with 140 and 108 cases per 100,000 inhabitants, respectively [3]. Mortality rates for dengue can be high when diagnosis and treatment are not appropriate, reaching values of 20% [4].

In 2009, World Health Organization (WHO) published guidelines for diagnosis, treatment, prevention and control of dengue [5]. These guidelines are, currently, used by medical personnel for the clinical management of dengue, from diagnosis to treatment of patients, and, used to avoid complications leading to death. However, there are still difficulties in the diagnosis and treatment of the disease. The main difficulty in these two aspects of dengue lies –mainly– in the large amount of information that the medical staff must analyze in a short time to define the procedure to follow for each particular patient. This information corresponds to demographic, clinical and laboratory variables such as age, signs and symptoms that a patient with dengue may present [6]. One way to address this problem is to use decision support systems (DSS) to support the decision-making of medical personnel caring for dengue patients. Such systems can use data to enhance the processes performed by a human being [7].

With respect to the previously presented background, the contribution of this paper is a clinical DSS using an autonomous cycle of data analysis tasks (ACODAT) to aid decision-making in clinical settings. In particular, ACODAT uses the interaction of different successive tasks to extract the necessary knowledge to recommend improvements in a given process [8]. The use of ACODAT in different fields such as education, telecommunications and industry 4.0, have been reported [9], [10], [11]. For example, in the educational field, ACODAT has been used to determine learning styles in smart classrooms. Aguilar et al. [9] used ACODAT to analyze web and social network data to build knowledge models about students. These models are used to permanently monitor the learning process. The results showed the capacity of ACODAT for the generation of useful knowledge to improve the learning process. In the field of telecommunications, Morales et al. [10] developed ACODAT for quality of service management in Internet of Things (IoT) platforms. The implemented ACODAT allowed analyzing the quality of IoT platforms using classification and clustering tasks. In Industry 4.0, ACODAT has been developed and implemented to improve the efficiency of production processes. For example, Sanchez et al. [11] presented a framework that helps to solve the problems of integration and heterogeneity of the actors involved in manufacturing processes. The results show that ACODAT allowed to these actors (people, data, things and services) to interact for the creation of a self-configuration and self-optimization plan. Finally, it also has been used in smart cities, to control and supervise heating, ventilation, and air conditioning systems [12], [13].

The ACODAT concept has not been applied in the field of medicine. Particularly, ACODAT has not been used for clinical disease management to date. Based on the problem of dengue, a disease that generates high mortality rates if not diagnosed or treated in time, and its economic impact on health systems, it is necessary to develop clinical DSS for the clinical management of dengue. For this reason, the objective of this work is to develop an ACODAT to support decision-making for the clinical management of dengue. Currently, there are different clinical DSS for dengue [14]; however, the studies reported in the literature use predictive and prescriptive approaches separately, and to date, there are no models that integrate these two approaches, which are closely related to each other. Especially, prediction alone is not very useful when there is no prescriptive model to recommend the best options for solving the problem. The main contribution of this work is the development and implementation of an ACODAT that verifies and corrects clinical data, classifies dengue patients and recommends the best treatment options to avoid complications and death of patients.

The remainder of this paper is structured as follows: Section 2 presents a brief literature review about dengue modeling for the clinical management of dengue. Section 3 introduces the generalities of dengue and the conceptualization of ACODAT. Section 4 describes the ACODAT proposed in this article, and the methodology used for its definition and implementation. Section 5 shows the results of ACODAT's implementation in two dengue datasets. Section 6 discusses the results and compares them with previous studies. Finally, Section 7 concludes the paper.

## 2 Related work

In this section, we show a brief literature review on dengue modeling for the clinical management of dengue. To date, many machine learning (ML) models have been developed to support dengue diagnosis (see [14] for more information). Here, we present the most recent ones related to early detection, classification of the disease and prescription of the treatment.

### 2.1 Early detection of dengue

Early detection of dengue is difficult and challenging due to the lack of specificity in the clinical presentation of the disease. However, in recent years, computer-aided strategies have been developed to support medical professionals in these difficult tasks. [15], [16]. For example, Khosavanna et al. [15] used two techniques, logistic regression (LR) and decision trees (DT), to develop predictive models for the assessment of possible early dengue infections. The authors used self-reported clinical manifestations from patients in non-endemic regions. The best performance was from the DT model with an area under the curve (AUC) of 0.75. Ho et al. [16] compared several ML techniques to identify confirmed dengue cases using only age, body temperature, white blood cell count and platelet count. Models were built with deep learning, DT and LR, where deep learning performed best with an AUC of 0.86.

### 2.2 Dengue classification

Dengue is classified into three types according to WHO: non-severe dengue (with or without warning signs) and severe dengue (SD). Differentiation of these stages can be difficult in some cases due to the variability of the signs and symptoms of dengue. Different studies have attempted to model this type of problem to support diagnostic decision-making [17], [18], [19]. For instance, Huang et al. [17] used demographic data and laboratory test results to classify dengue patients based on its severity. Several ML methods such as LR, random forest (RF), support vector machines (SVM) and artificial neural networks (ANN) were used to train the models. The best model was ANN with an accuracy of 0.75.

Chatterjee et al. [18] proposed a hybrid ANN model with a modified cuckoo optimization algorithm. The model proposed by Chatterjee et al. had an accuracy of 0.957 using gene expression data. However, the classification performed was based on that recommended by WHO in 1997 (dengue fever, dengue hemorrhagic fever and dengue shock syndrome) [20].

Hoyos et al. [19] developed a DSS for dengue using fuzzy cognitive maps (FCM). They implemented diagnostic models using FCM to classify patients according to the type of dengue, with an accuracy of 0.89. Also, they analyzed the behavior of signs, symptoms, laboratory tests and disease severity. This study goes further, and not only classifies the patient, but also evaluates the behavior of the signs and symptoms of dengue over time, giving recommendations as to what factors might influence and appear in the course of the disease.

### 2.3 Dengue treatment

Treatment of dengue consists of palliating symptoms and avoiding complications leading to death. The complexity of the treatment is represented by the high variability of the clinical manifestations presented. Despite WHO recommendations, the treatment of dengue remains a challenge for medical professionals. Unfortunately, to date, no computational models have been developed to support decision making regarding the treatment of dengue.

In summary, the approaches proposed for the diagnosis of dengue based on severity are few. The models developed by [15] and [16] have the limitation of only detecting the disease without classifying it. On the other hand, the approaches developed for the classification of dengue have limitations such as the low classification performance in the work of [17], or the use of genetic data by [18], which is not useful in clinical practice because this type of data is not easy available for the clinician. Finally, there are no prescribing approaches that recommend treatment options for dengue.

The clinical management of dengue comprises both diagnosis and treatment. Thus, there is a need for the development of prescriptive models (treatment) integrated with classification models (diagnosis) to support decision making. The use of clinical data such as signs, symptoms and routine laboratory tests for the development of these models is important because of the availability and ease of collection in regular clinical settings.

## 3 Theoretical background

### 3.1 Clinical management of dengue

In this section, we describe the principal aspects of dengue, including generalities, diagnosis and recommendations for treatment.

#### 3.1.1 Generalities of dengue

Dengue is an acute infection caused by a virus of the flavivirus group. To date, there are four (4) serotypes of the virus (DENV-1, DENV-2, DENV-3 & DENV-4). The infection is transmitted from person to person by the bite of an *Aedes* mosquito [21]. Dengue can be classified according to the severity of the disease into: 1) non-severe dengue without warning signs (NoWS-Dengue), 2) non-severe dengue with warning signs (YesWS-Dengue) and, 3) SD. This classification was recommended by a WHO expert group in 2009 [5]. Dengue has various forms of clinical expression: undifferentiated fever, headache, general malaise, osteomyoarticular pain, with or without exanthema and leukopenia. Severe forms of the disease are characterized mainly by hypovolemic shock caused by plasma extravasation, with moderate or severe thrombocytopenia and major bleeding in the gastrointestinal tract and other locations [22]. Dengue is also capable of expressing itself through the so-called “atypical” forms, which are relatively infrequent and result from particularly intense involvement of an organ or system: encephalopathy, cardiomyopathy or hepatopathy, among others [23].

#### 3.1.2 Diagnosis of dengue

The definitive and confirmatory diagnosis of dengue is made using direct methods such as virus isolation, detection of viral nucleic acid or antigens; and indirect methods such as detection of antibodies produced against the virus [2]. However, these laboratory tests can take a long time, which could cause the patient with dengue to develop complications and die. To solve this problem, there are dengue diagnosis guidelines published by WHO [5]. These guidelines state that the first step in the diagnosis of dengue is the general evaluation of the patient by the physician to classify the patient into a group: NoWS-Dengue, YesWS-Dengue & SD. The physical examination, analysis of the medical history, and laboratory tests such as a complete blood count, allow the identification of warning signs and evaluation of the patient's hydration status. Classification of the patient into a group constitutes the second stage in the clinical management of dengue. The use of this guide is crucial to provide adequate management of the disease due to the wide spectrum of clinical manifestations of dengue.

#### 3.1.3 Recommendations for treatment

The third step in the clinical management of dengue is treatment. The information obtained in the previous two steps is vital to provide an adequate and timely treatment for the patient with dengue. Table 1 summarizes the clinical management of dengue, by treatment group, based on the WHO guidelines. The treatment routes for dengue are categorized into three groups (A, B & C). In group A, we have patients who do not present warning signs or comorbidities and who tolerate oral water volumes. In addition, this group includes patients with adequate diuresis. In group B, we have patients with warning signs or pre-existing conditions such as diabetes mellitus, obesity, renal failure, pregnancy, among others. Patients with some social conditions, such as living alone or living far from a health institution, are also classified in this group. Finally, group C constitutes all patients with any of the following complications: severe plasma extravasation, severe bleeding, shock, and severe organ deterioration.

**Table 1:** Summary of clinical management of dengue by treatment group recommended by WHO [5].

Treatment group	Characteristics	Management
*A*	No warning signs	Paracetamol
	Tolerate adequate volumes of oral fluids	Drink water
	Adequate diuresis	Oral intake of rehydration solutions
	Normal hemogram	Daily monitoring

*B*	Warning signs	Hospitalization
	Comorbidities	Isotonic solutions
	Social conditions	Hematocrit and platelets monitoring

*C*	Severe extravasation	Hospitalization
	Severe bleeding	Isotonic solutions 5-7 ml/kg/hour
	Shock	Colloid solutions 10-20 ml/kg/hour
	Organ failure	Vital signs monitoring

### 3.2 ACODAT

The high amount of data generated today continues to increase considerably. For this reason, it is necessary to develop new tools for data manipulation to extract meaningful knowledge. ACODAT is one of these strategies, which consists of a set of data analysis tasks that must be performed together to achieve an objective in a given system or situation [24]. This set of tasks interacts, and has different roles in the cycle [25], [26]: observing the process, analyzing and interpreting what happens in it, and making decisions to achieve the objective for what the cycle was designed.

The performance of successive tasks connected allows solving complex problems that require a lot of knowledge for the solution [8]. The tasks in an ACODAT can be classified into three types [27]: observation, analysis and decision making. Observation tasks are those in charge of collecting data and information about the system or environment. The analysis tasks are in charge of interpreting or diagnosing the system using data. This function is performed by building knowledge models about the behavior of the cycle. Finally, the decision-making tasks are those in charge of performing decision-making activities to improve the process.

## 4 Methodology

In this section, we describe the methodology to create an ACODAT for the clinical management of dengue. Then, we specify each of ACODAT's tasks. Finally, we show the implementation of these tasks on specific datasets to support decision-making related to clinical management of dengue.

**Figure 1:**
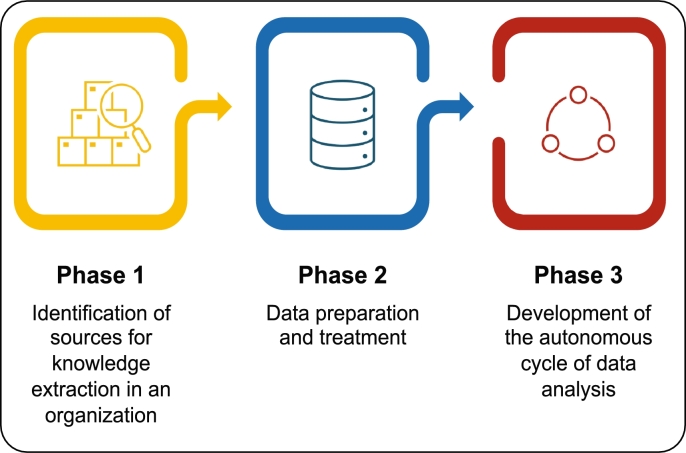
MIDANO methodology. Adapted from [29].

MIDANO is a *methodology for data analytic based on organizational characterization* that has been defined for the development of applications based on data analysis, and especially, ACODAT [28]. In this paper, we use the MIDANO methodology with a little modification for the development of this work. The MIDANO methodology consists of three main phases (see Fig. 1). The objective of phase 1 is to know everything related to the organization to define the objective of the data analysis application. This stage focuses on identifying and conceptualizing the solution to a problem, from the perspective of developing applications based on data analysis. Phase 2 is in charge of data preparation and treatment. This process is based on the ETL paradigm (E = Extraction of data from its sources, T = Transformation of data, and L = Loading of data). The main objective of this stage is to generate quality data in order to create knowledge models, and define the multidimensional data model of ACODAT. The objective of phase 3 is the implementation of the data analysis tasks in the ACODAT to generate knowledge models (descriptive, predictive, classification, prescriptive, among others) [29]. In our work, the first phase was used to characterize the problem. In addition, we included data preparation and treatment (second phase) inside the ACODAT, such a way that the data is processed online (real-time) by the cycle to make the process more autonomous. Also, the second phase was responsible to identify the data sources needed to build the ACODAT. Next, we explain –in detail– each of the MIDANO phases applied to the clinical management of dengue.

### 4.1 Characterization of the dengue context

The first phase of the MIDANO methodology is to identify sources to extract knowledge in an organization [29]. In this case, we met with dengue clinical experts to identify those knowledge sources. The clinical experts in dengue expressed the difficulties presented in the hospital environment: 1) problems with the labeling of patients with each type of dengue due to unintentional errors in the database entry, 2) difficulties in the classification of patients with dengue due to the high number of variables to analyze in a short time, added to this, the lack of experience of some physicians for a correct classification of the patient with dengue, and, 3) difficulties in the palliative treatment of dengue for the same reasons expressed above.

### 4.2 Identification and analysis of data sources

The second phase of MIDANO corresponds to the identification of data sources that can help develop clinical DSS for dengue. In this case, the most suitable option is the use of open databases published by the Colombian government through the National Institute of Health (INS in Spanish). The data that health institutions report to the Colombian national health institute were identified. These data correspond to demographic variables such as age, clinical variables such as signs and symptoms, and finally, results of laboratory tests. In MIDANO methodology, this phase also considers the preparation and treatment of the data; however, to make the process more dynamic, we included the processing of the data within the ACODAT.

### 4.3 Specification of the ACODAT for the clinical management of dengue

**Figure 2:**
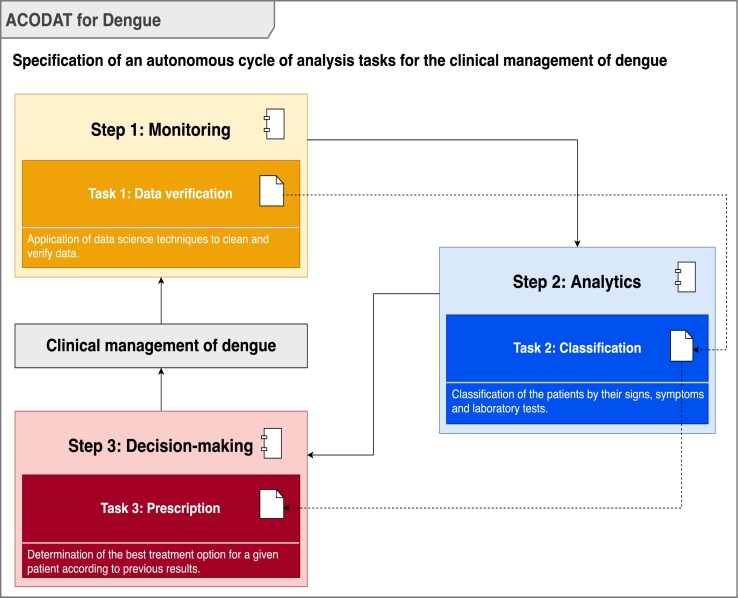
Architecture of ACODAT for clinical management of dengue.

The last phase of MIDANO corresponds to the specification of ACODAT. This paper proposes an ACODAT for the clinical management of dengue. Fig. 2 shows the architecture of ACODAT for clinical management of dengue. This ACODAT is composed of three steps with interconnected tasks for the improvement of dengue decision-making at the hospital level. Step 1, called *monitoring*, comprises the tasks of data verification and correction. Step 2, called *disease analysis*, consists of the task of classifying patients based on their signs, symptoms and laboratory tests. Finally, step 3, called *treatment decision making*, comprises the prescription task, which consists of recommending the best treatment option for a given patient. The data analysis tasks used techniques that belong to different fields of artificial intelligence (AI), such as ANN and genetic algorithm (GA) which belong to the field of computational intelligence; and SVM which belongs to the ML field.

#### 4.3.1 Task 1: data verification and correction

The results obtained in modeling depend largely on the quality of the data [30]. The first task of our ACODAT is to detect and correct possible errors to perform the next tasks in the best way. Missing data is very common in this kind of data; for this reason, rows with missing data are removed from the dataset. Another problem with dengue datasets is the imbalance of their classes, because one of the classes (SD) is always in lower proportion to the other two classes (NoWS-Dengue & YesWS-Dengue). For this reason, an oversampling technique was used.

#### 4.3.2 Task 2: classification

After data are prepared and verified, they are passed to the second ACODAT task. This task uses classification techniques to determine the type of dengue in patients. For this task, we used ANN and SVM. The main characteristics of this task are shown in Table 2.

**Table 2:** Description of the ACODAT's tasks for clinical management of dengue.

Task name	Characteristics of the task				
	Description	Data source	Analytics type	Technique	Knowledge model
*Data verification*	Verification of data and correction of errors	Datasets of National Institutes of Health about dengue	Description	Verification Oversampling	Descriptive
*Classification*	Classification of a patient by their variables	Previous task	Classification Prediction	ANN SVM	Predictive
*Prescription*	Determination of the best treatment option for dengue	Previous task	Optimization	Genetic algorithm	Prescriptive

#### 4.3.3 Task 3: prescription

This task uses a list of prescriptions for dengue –described in the WHO guidelines– for the clinical management of dengue. Based on the results of the previous task, a GA optimizes the best treatment option for a particular patient. Table 2 shows the characteristics of this task.

### 4.4 Implementation of ACODAT for the clinical management of dengue

In this section, we implemented ACODAT for the clinical management of dengue using datasets from two regions of Colombia.

#### 4.4.1 Datasets

The Data used for this implementation are those stored in the database of the Colombian epidemiological surveillance system (SIVIGILA in Spanish), which correspond to records of dengue patients reported by health institutions to the Colombian National Health Institute, the entity in charge of managing this type of information in Colombia. For the experiments, we used data from the city of Medellin (2008-2018) and the department of Córdoba (2010-2021) [31]. We chose these regions because they are endemic for dengue. According to epidemiological reports, the annual incidences reported are 161-745 and 51-503 per 100,000 inhabitants for Medellín and Córdoba, respectively [32].

Medellín and Córdoba datasets were composed of 52,051 and 16,670 patients, respectively. Both datasets had 36 variables, of which 14 were eliminated because they did not contribute to our study or were not related to the clinical management of dengue, for example: address, type of social security, city and department codes, among others. Finally, 22 variables were selected corresponding to the signs, symptoms and laboratory tests that the medical professional observes or detects in each patient suspected of dengue. Table 3 shows each of the variables included in the datasets, their type and a brief description. All predictor variables in the datasets are binary (except age, which was numerical), where 1 represents the presence of the sign or symptom and 0 represents the absence. The target variable is categorical with 3 classes corresponding to the WHO classification of dengue (No WS-Dengue, Yes WS-Dengue & SD).

**Table 3:** Variables used to build the ACODAT for clinical management of dengue.

Code	Variable	Type of variable	Description
V1	Age	Demographic	Time elapsed since the birth of an individual
V2	Fever	Sign/symptom	Increase in body temperature
V3	Cefalea	Symptom	Pain and discomfort located in any part of the head
V4	Pain BE	Symptom	Pain behind eyes
V5	Myalgias	Symptom	Muscle aches
V6	Arthralgias	Symptom	Joint pain
V7	Rash	Sign/symptom	Skin exanthema
V8	Abd. pain	Sign/symptom	Intense pain, located in the epigastrium and/or right hypochondrium
V9	Vomit	Symptom	Violent expulsion by the mouth of what is contained in the stomach
V10	Lethargy	Sign/symptom	State of tiredness and deep and prolonged sleep
V11	Hypotens.	Sign	Excessively low-blood pressure on the artery wall
V12	Hepat.	Sign	Condition of having an enlarged liver
V13	Muc. hemo.	Sign/symptom	Manifestations of mild to severe bleeding in the nasal mucosa, gums, skin, female genital tract, brain, lungs, digestive tract and hematuria
V14	Hypoterm.	Sign/symptom	Decrease of body temperature
V15	High hem.	Lab. test	Indirect increase in hematocrit test
V16	Low plat.	Lab. test	Decrease of platelet levels in the blood
V17	Edema	Sign/symptom	Swelling caused by excess fluid trapped in body tissues
V18	Extrav.	Sign	It is characterized by serous spills at the level of various cavities
V19	Bleeding	Sign/symptom	Blood leaks from the arteries, veins or capillaries through which it circulates, especially when it is produced in very large quantities
V20	Shock	Sign	Manifestation of severity evidenced by cold skin, thready pulse, tachycardia and hypotension
V21	Org. fail.	Sign	Affectation of several organs due to the extravasation of liquids
V22	Dengue category	Target	Type of dengue based on the severity

#### 4.4.2 Implementation of task 1: data verification

In general, health data have some very common particularities, such as low quality [30]. In the case of dengue data, there are many errors for different reasons. One of the reasons is the speed of the medical professionals in entering the dengue notification forms to the health authorities. Also, the high demand for hospital care causes medical professionals to enter unintentional errors into the datasets. For example, a very common error in the databases is to find patients with NoWS-Dengue classified as having SD.

Missing data treatment was carried out using the listwise method, which consists of eliminating all the data of an observation if there is at least one missing data. For class balancing, we used the Synthetic Minority Oversampling Technique (SMOTE) due to the low frequency of the SD category. Table 4 shows the distribution of dengue type in the datasets after applying preprocessing techniques.

**Table 4:** Distribution of dengue categories in the datasets.

Dataset	Dengue category	Original	After listwise deletion	After balancing
Medellín	No WS-Dengue	27,230	10,210	10,210
	Yes WS-Dengue	12,669	11,123	11,123
	SD	437	123	11,186
	Total	52,051	21,456	32,519

Córdoba	No WS-Dengue	9,905	4,563	4,563
	Yes WS-Dengue	6,179	5,134	5,134
	SD	586	231	5,623
	Total	16,670	9,928	15,320

**Figure 3:**
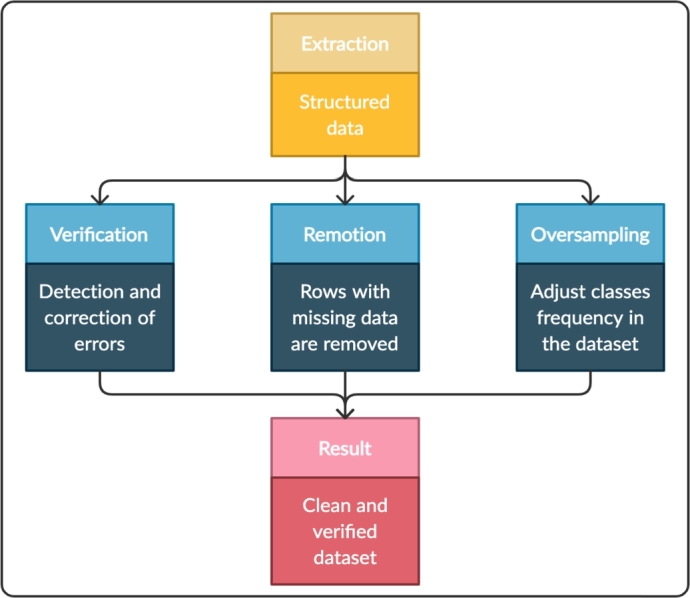
Activities or sub-tasks related to task 1 (data verification and correction).

For this first task, a Python 3.5 program was written to verify and correct the data. We used libraries such as *Pandas* [33] to extract and process the structured data. The *Imbalanced-learn* library [34] was used to correct the imbalance of the classes. The steps to follow in this task are the following: 1) extract the structured database of patients with the three types of dengue, 2) verify if there are errors in the patient labels; for example, if there are patients without warning signs classified as SD, reassign the label as NoWS-Dengue, 3) eliminate rows with missing data because the value of the variable for that patient cannot be established, 4) Balance the classes using the oversampling technique that consists of increasing the number of samples of lower frequency in the dataset [35]. In this case, the records for the SD class. Fig. 3 represents the activities or subtasks performed in this task.

#### 4.4.3 Implementation of task 2: classification

**Figure 4:**
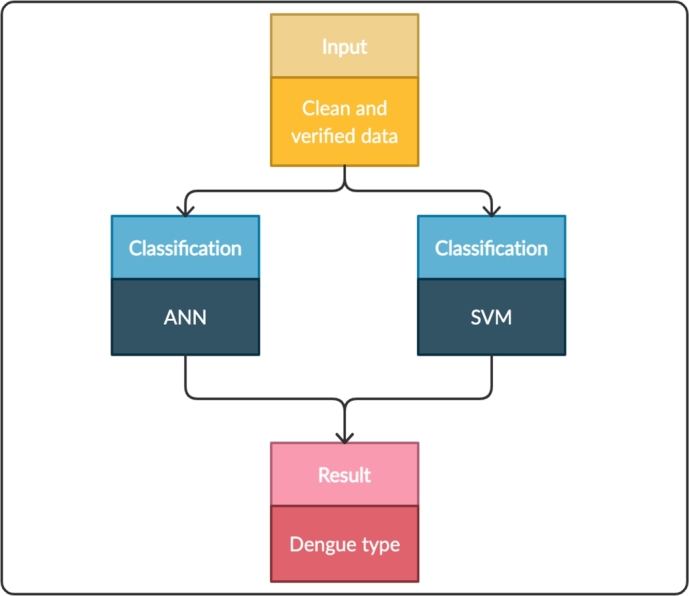
Steps related to task 2 (classification).

The second task of the ACODAT classified the patients in the three labels of the dataset. Fig. 4 shows the activities in this task. The labels were NoWS-Dengue, Yes-WS-Dengue & SD. This task was performed using ANN and SVM techniques, which were chosen for their high performance for classification with clinical datasets [19].

This task had as input the clean and verified dataset product of the previous task (data verification and correction). We divide the dataset in 70% for training and validation, and 30% for testing. We used 10-fold cross-validation to find the best combination of hyperparameters and used different configurations for both ANN and SVM. In the case of ANN, we used a multi-layer perceptron with a single layer, and for SVM, we used SVM in its classifier version. Table 5 shows the different configurations of hyperparameters for each implemented technique.

**Table 5:** Hyperparameter settings used to build the ANN and SVM models.

Technique	Hyperparameter	Options
*ANN*	Number of hidden units	16, 32, 64, 128, 256
	Learning rate	0.0001, 0.001, 0.01, 0.05, 0.1, 0.5
	Activation function	tanh, ReLU
	Optimizer	Gradient descent, Adam

*SVM*	Kernel	Linear, radial, sigmoid
	C	0.0001, 0.001, 0.01, 0.1, 1.0, 10.0, 100.0, 1000.0
	gamma	0.0001, 0.001, 0.01, 0.1, 1.0, 10.0, 100.0, 1000.0

The implementation of this task was performed in Python 3.5 using the *Scikit-learn* library [36] for modeling and the *Numpy* library [37] for matrix and vector processing.

#### 4.4.4 Implementation of task 3: prescription

**Figure 5:**
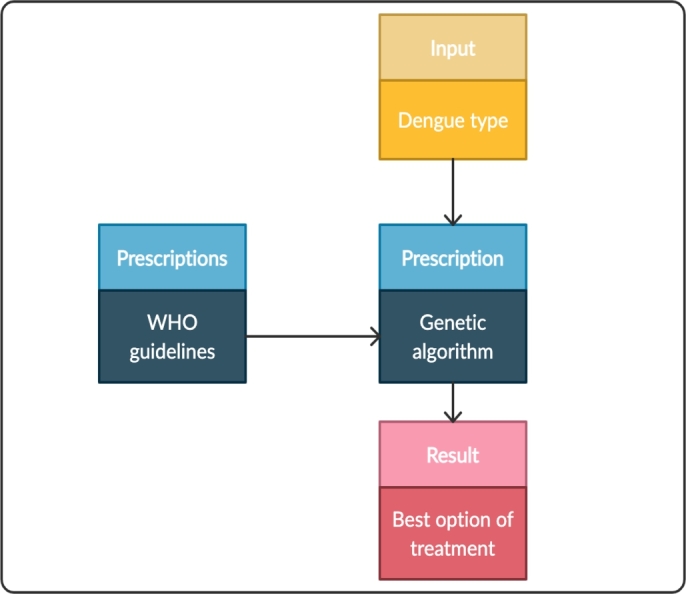
Steps related to task 3 (prescription).

Task 3 was focused on decision-making. In this case, it was focused on determining the best treatment option for a patient with a particular type of dengue. Fig. 5 shows the steps of this task. The implementation of this task was performed using a GA to find an optimal solution to the problem. The first step of this task was to identify some prescriptions recommended by WHO in the guidelines for the treatment of dengue. We identified six prescriptions: paracetamol (*P*), drinking water (*W*), oral rehydration solutions (*ORS*), isotonic solutions 5-7 ml/kg/hour (*IS*), colloid solutions 10-20 ml/kg/hour (*CS*) and hospitalization (*H*). These are the main prescriptions recommended by WHO for the treatment of dengue (see Table 1 and [5] for more information).

**Figure 6:**
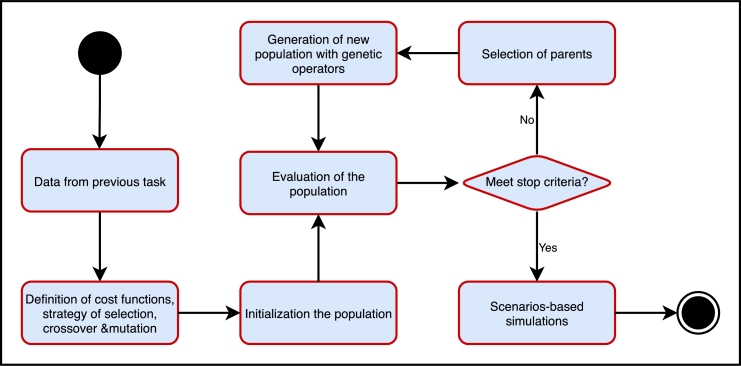
Methodological flowchart to create a prescriptive model using a GA.

**Figure 7:**
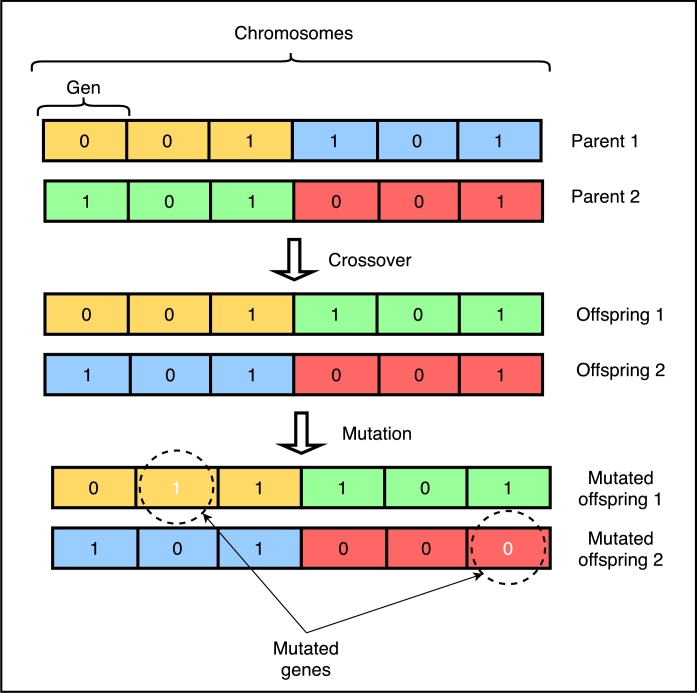
Example of chromosomes, crossover and mutation processes in a GA.

Fig. 6 summarizes the methodological framework for the creation of a prescriptive model using a GA. The prescriptions were binary coded to feed the GA. The steps to find the optimal solution were: 1) generation of random binary chromosomes representing different solutions (alternative prescriptions). The number of generations depended on having individuals with prescriptions greater than or equal to 95% assertiveness. 2) estimation of the fitness of each chromosome using a function, 3) creation of new individuals using genetic operators. In this step, two parent chromosomes with the best fitness are selected, and crossover and mutation operators are used in them. 4) generation of a new population for a new iteration/generation. Fig. 7 shows a graphical representation of the chromosomes, crossover and mutation processes. The crossover and mutation probabilities were set to 0.5, respectively.

Several studies have shown that these probability values generate the best performance results on similar problems [38], [39], [40]. The crossover operator takes two selected parents and cuts the chromosomes at a randomly chosen position to produce two initial and two final gene subsets. The final subsets are then swapped, producing two new complete chromosomes. The mutation operator is applied to each offspring individually, and consists of the random alteration of each component gene of the chromosome. Regarding the fitness functions to evaluate the possible solutions, these were established based on the type of dengue. All the fitness equations proposed have as output a value between 0 and 100 that corresponds to the fitness of a solution (chromosome) to solve the problem, being 0 not suitable at all and 100 very suitable. The variables involved in these functions are: the list of prescriptions described above (*P*, *W*, *ORS*, *IS*, *CS*, *H*), $WP_{i},\left\{i=1,2,3\right\}$, which corresponds to the penalty when unsuitable treatment options are recommended for the type of dengue, $F_{i}$ which corresponds to the result of the fitness function for each type of dengue. The *ith* value corresponds to the dengue type. Finally, the *r* value is a random number to increase the searching space.

(1) $$WP_{1}=IS\times 2+CS\times 2$$

(2) $$F_{1}=(r(30,40)\times P+r(50,60)\times W)\times 0.8^{WP_{1}}\times 0.1^{H}\;$$

(3) $$WP_{2}=P+CS\times 2$$

(4) $$F_{2}=(r(65,75)\times IS+r(15,25)\times ORS)\times 0.8^{WP_{2}}\times 0.1^{1-H}\;$$

(5) $$WP_{3}=P+W\times 2$$

(6) $$F_{3}=(r(65,75)\times CS+r(15,25)\times IS)\times 0.8^{WP_{3}}\times 0.08^{1-H}\;$$

All fitness equations were constructed with the help of dengue clinical experts, who assigned the coefficients for each variable or treatment option depending on its importance in the clinical management of the disease for each type of dengue. Eq. (2) is the fitness function for NoWS-Dengue. In this type of dengue, the use of recommendations such as the application of IS and CS is not recommended, so the fitness function penalizes the use or the presence of this recommendation in a chromosome during the optimization process (see Eq. (1)). For this case, the clinical experts in dengue assigned a coefficient of 0.8 to penalize the use of these strategies in this type of patient. In addition, the fact of being hospitalized (H) is penalized, because a patient with no warning signs does not need to be hospitalized [5]. For this case, the experts assigned a coefficient of 0.1 for this variable, because it is not so serious for the patient to be hospitalized. Random intervals are included to simulate the fact that prescriptions are not absolute and a prescription will not work the same for all patients. In these random intervals, a little more weight is given to W since hydration is key to keeping the patient from getting worse [5]. In Eq. (4) corresponding to YesWS-Dengue, the use of CS is penalized because they are patients who are not in severity and do not require this type of treatment (see Eq. (3)). Besides, there is a severe penalty if the patient is not hospitalized, since patients with warning signs need to be closely monitored given the high risk of worsening [5]. In the same way as in Eq. (2), experts assigned coefficients for each treatment variable. Finally, Eq. (6) represents the fitness function for SD. In this function, the use of P and W is penalized (see Eq. (5)) because these patients are in a state of severity and do not tolerate the use of oral solutions or medications. Finally, it is penalized if the patient is not hospitalized, since patients with SD need to be treated on an emergency basis [5]. In the same way as in Eq. (2) and Eq. (4), experts assigned coefficients for each treatment variable to build fitness functions.

Due to the lack of datasets with dengue treatment results, we implemented this prescriptive task in some specific scenarios. We use a binary vector to represent the patient's age, clinical and laboratory variables, where 0 means the absence of the variable and 1 means the presence of the variable. For the type of dengue, we used 1 = NoWS-Dengue, 2 = YesWS-Dengue and 3 = SD. With respect to treatment options, 1 means that this treatment option is recommended for that patient, while 0 represents that this treatment option is not recommended. The implementation of this task was done using the Python 3.5 *Pandas* and *Numpy* libraries [33], [37].

## 5 Results

In this section, we show the results of each of the tasks implemented in ACODAT. First, we show the main characteristics of the cleaned and corrected datasets. Second, the results of the classification model and, finally, the results of the prescription in specific scenarios.

### 5.1 Clean and corrected dataset

The first task aims to detect and correct errors in the dataset. Table 4 shows the results of the dataset after applying different data science techniques for data correction. Finally, we used 32,519 and 15,320 records to generate the classification models for Medellín and Córdoba, respectively.

**Figure 8:**
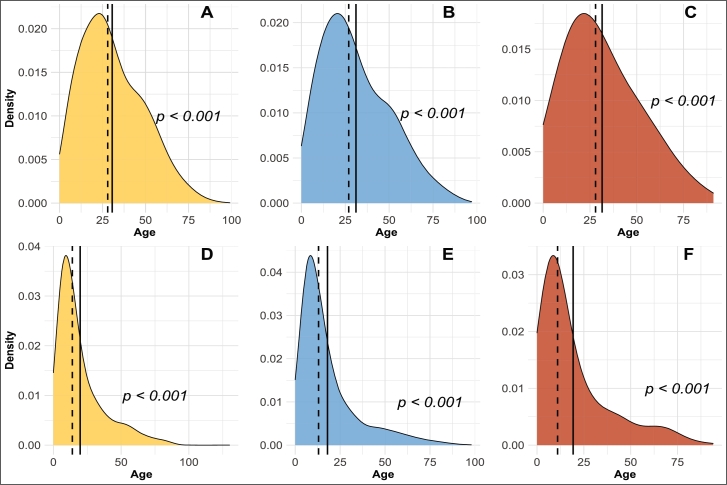
Age distribution in the datasets according to the dengue category (yellow = NoWS-Dengue, blue = YesWS-Dengue, red = SD). **A**, **B** and **C** correspond to the Medellín dataset, while **D**, **E** and **F** correspond to the Córdoba dataset. The solid and dash lines indicate the mean and the median, respectively. The p-values are the result of normality test for each class.

The description of the categorical variables in the dataset was done in the previous section. The only quantitative variable was age, and its distribution is shown in Fig. 8. Plots A, B and C in Fig. 8 represent the age distribution in the Medellín dataset, while plots D, E and F represent the age distribution in the Córdoba dataset. The distribution of age, in the three dengue categories, showed similar results for both the Medellín and Córdoba datasets. The Kolmogorov-Smirnoff test was performed to test the normality of this variable. The results showed that they do not follow a normal distribution (p<0.001 for the three categories). The right-skewed density curves in Fig. 8 confirm the results, where the average age is greater than the median.

### 5.2 Classification models

**Figure 9:**
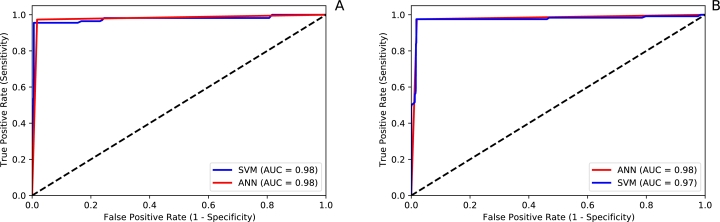
ROC curves to evaluate the quality of the models used to classify dengue patients. **A** = Medellín dataset, **B** = Córdoba dataset.

Table 6 and Fig. 9 show the performance results of this task, and the optimal values of the hyperparameters for each technique. Plot A in Fig. 9 corresponds to the performance of the classification model on the Medellin dataset, while Plot B represents the performance on the Córdoba dataset. The implemented models showed an excellent performance to classify patients based on severity. The best performing model was the one developed with the Medellin dataset, with an accuracy of 0.981 and AUC of 0.98. However, all models had a high performance with accuracies above 0.97.

**Table 6:** Quality of developed models used to classify dengue patients.

Model	Hyperparameters	Dataset			
		Medellín		Córdoba	
		Accuracy	F1-Score	Accuracy	F1-Score
ANN	• 256 hidden units	0.979	0.978	0.977	0.977
	• ReLU				
	• Adam				
	• *α* = 0.01				

SVM	• Radial kernel	0.981	0.981	0.972	0.971
	• C = 10				
	• *γ* = 10

### 5.3 Prescriptive model

In this section, we show the results of the prescriptive model in specific scenarios. We chose three scenarios (one for each type of dengue) to show the ability of the model to prescribe treatment for dengue in each disease variant.

#### 5.3.1 Scenario 1

Patient 65 years old with following symptoms: fever, headache, myalgias. The previous task (classification) classifies this patient as NoWS-Dengue. This information is received by the prescriptive task, and based on the fitness function assigned for this type of dengue (Eq. (2)), it optimizes the solution that corresponds to the best treatment option for this patient. Table 7 shows the vectors corresponding to the patient variables, the result of the classifying task, and the result of the prescriptive task showing the options that are recommended for this particular patient. In this case, the output of the predictive model is used by the prescriptive model to optimize the optimal treatment for this patient.

**Table 9:** Results of classification and prescription tasks for a patient with NoWS-Dengue. Results of classification and prescription tasks for a patient with YesWS-Dengue. Results of classification and prescription tasks for a patient with SD.

Variables (age, signs, symptoms and laboratory tests)																					Dengue type	Treatment options					
V1	V2	V3	V4	V5	V6	V7	V8	V9	V10	V11	V12	V13	V14	V15	V16	V17	V18	V19	V20	V21	V22	P	W	ORS	IS	CS	H
1	1	1	0	1	0	0	0	0	0	0	0	0	0	0	0	0	0	0	0	0	-	-	-	-	-	-	-
↓																											
Classification task																											
↓																											
1	1	1	0	1	0	0	0	0	0	0	0	0	0	0	0	0	0	0	0	0	1	-	-	-	-	-	-
↓																											
Prescription task																											
↓																											
1	1	1	0	1	0	0	0	0	0	0	0	0	0	0	0	0	0	0	0	0	1	1	1	1	0	0	0



Variables (age, signs, symptoms and laboratory tests)																					Dengue type	Treatment options					
V1	V2	V3	V4	V5	V6	V7	V8	V9	V10	V11	V12	V13	V14	V15	V16	V17	V18	V19	V20	V21	V22	P	W	ORS	IS	CS	H
0	0	1	0	1	1	0	1	1	0	0	0	0	0	0	0	0	0	0	0	0	-	-	-	-	-	-	-
↓																											
Classification task																											
↓																											
0	0	1	0	1	1	0	1	1	0	0	0	0	0	0	0	0	0	0	0	0	2	-	-	-	-	-	-
↓																											
Prescription task																											
↓																											
0	0	1	0	1	1	0	1	1	0	0	0	0	0	0	0	0	0	0	0	0	2	1	1	1	1	0	1



Variables (age, signs, symptoms and laboratory tests)																					Dengue type	Treatment options					
V1	V2	V3	V4	V5	V6	V7	V8	V9	V10	V11	V12	V13	V14	V15	V16	V17	V18	V19	V20	V21	V22	P	W	ORS	IS	CS	H
0	1	0	0	1	1	0	0	0	0	0	0	0	0	0	0	0	0	0	1	0	-	-	-	-	-	-	-
↓																											
Classification task																											
↓																											
0	1	0	0	1	1	0	0	0	0	0	0	0	0	0	0	0	0	0	1	0	3	-	-	-	-	-	-
↓																											
Prescription task																											
↓																											
0	0	1	0	1	1	0	1	1	0	0	0	0	0	0	0	0	0	0	0	0	3	0	0	0	1	1	1

The results show that the prescriptive model recommends the use of P, W and ORS. This result is correct because, in this patient, it is only important to rehydrate to maintain plasma volume and P to relieve symptoms such as fever, headache and muscle aches. Regarding the other decision variables, it is not necessary to apply IS or CS because there are no signs indicating fluid accumulation in the patient's body. Furthermore, this type of patient does not need to be hospitalized, so the prescriptive model does not recommend this treatment option. In summary, the prescriptive model makes a correct recommendation with respect to the WHO recommendations.

#### 5.3.2 Scenario 2

Patient 35 years old with: headache, myalgias, arthralgias, vomiting, abdominal pain. Using the result of the previous task, this patient is classified as *YesWS-Dengue*. The GA uses the fitness function of Eq. (4) to choose the best solutions for this particular patient. A chromosome with the best fitness is obtained. In Table 8, we can observe the age, signs, symptoms and laboratory tests of this patient represented in a vector. In addition, we can observe the type of dengue classified by the previous task, and, finally, we observe the best treatment options for this patient. In this scenario, the most important finding is that the patient presents two warning signs, such as vomiting and abdominal pain. Based on these findings, the prescriptive model recommends P, W, ORS, application of IS and H.

The presence of fever and pain in the patient confirms the recommendation of analgesics such as P. The use of ORS and W is recommended in this type of patient, since hydration is an important aspect to prevent dengue complications. However, as this patient presents some warning signs, such as vomiting and abdominal pain, the application of IS is necessary to help with the patient's hydration. Regarding hospitalization, the model prescribes that it is one of the best treatment options, due to the patient's warning signs. This type of patient must be constantly monitored and assessed to avoid complications and death. In summary, the prescriptive model developed makes a correct recommendation with respect to the clinical management guidelines for dengue published by WHO.

#### 5.3.3 Scenario 3

Case 3: Patient 49 years old with: fever, myalgias, arthralgias, shock. Using the result of the previous task, this patient is classified as having SD. The GA uses the fitness function of Eq. (6) to choose the best solutions for this particular patient. In Table 9, we can observe the age, signs, symptoms and laboratory tests of this patient represented in a vector. In addition, we can observe the type of dengue classified by the previous task, and, finally, we observe the best treatment options for this patient. In this case, the most important manifestation of the patient is shocked.

The use of P, W and ORS are not the best options. The prescriptive model does not recommend any of these options because it does not find them feasible for this patient. Instead, the prescriptive model recommends the application of IS and CS to restore the patient's plasma volume. In addition, the prescriptive model recommends hospitalization, since this patient should be hospitalized immediately for adequate treatment and follow-up. In summary, the prescriptive model recommends optimal and feasible treatment options for patients with SD. The recommendations made by the prescriptive model are in accordance with the recommendations published by the WHO.

## 6 Discussion

The clinical management of dengue is of vital importance to reduce mortality rates from the disease. Diagnosis and treatment must be optimal and prompt to avoid complications leading to death. We set out to develop an ACODAT to support decision-making in the clinical management of dengue.

Our proposal monitored data quality and corrected possible errors related to missing data, misclassification of dengue patients, and balancing of dengue categories. The quality of the models depends to a large extent on the quality of the data. The excellent quality of the classification models obtained in ACODAT's task 2 indicates the quality of the data used to train these models. Although in recent years data-driven strategies continue to increase, this aspect remains a challenge for modeling in medicine.

Dengue classification was performed using two ML techniques widely used in the medical field. ANN and SVM are excellent techniques for finding linear and nonlinear variable relationships in medical datasets. Few works have been developed using SIVIGILA datasets for dengue classification. The work of Hoyos et al. [19] developed a classification model using FCM. The results of this work showed an accuracy of 0.89. The results of our model showed a higher performance (see Table 6 and Fig. 9), perhaps because the relationships were extracted from the data and not assigned by experts, as occurs with FCM.

On the other hand, to date, there is no specific treatment for dengue. However, WHO has published treatment guidelines to alleviate symptoms and avoid complications. The non-specificity of signs and symptoms makes it difficult to choose the appropriate treatment in specific scenarios. The development of computer-aided strategies could support decision-making in clinical settings. In this sense, our work is the first study to report a prescriptive model to generate treatment recommendations based on WHO guidelines. The prescriptive model developed has the capacity to prescribe suitable actions for the palliative treatment of dengue.

We qualitatively compare our work with other similar works using some criteria listed below: A) The proposed approach uses AI techniques for the classification of dengue. B) The proposed approach uses a technique of AI to recommend the best option for the treatment of dengue. C) The proposed approach automates the clinical management of dengue (diagnosis and treatment). D) The proposed approach is intuitive, extensible y easily adaptable (e.g., if it can become a multi-agent clinical decision-making system [41]).

Table 10 shows the comparison between previous works and our research. The study by Chatterje et al. [18] implemented a hybrid approach to dengue classification using gene expression data. The authors used an ANN enhanced with the Cucko search optimization algorithm. The type of ANN used was the multi-layer perceptron with a single hidden layer in its structure. The aim of this work was to classify patients into different dengue classes; however, 2009 WHO dengue classification was not used. The authors used 1997 WHO dengue classification, which proposed to classify dengue into classic dengue, dengue hemorrhagic fever and dengue shock syndrome. Additionally, the data used were genetic, which is not easy or inexpensive to collect in routine clinical practice. Finally, this work does not present treatment options for the disease.

**Table 10:**
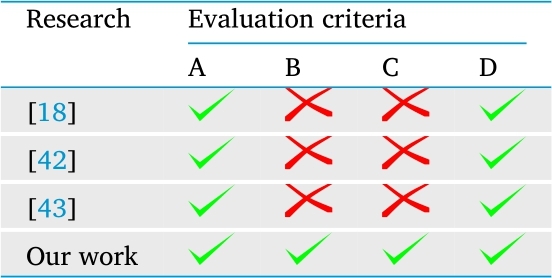
Criteria for evaluation of our work with previous works.

Macedo-Hair et al. [42] analyzed the clinical profiles of dengue patients to identify clusters of patients, and thus classify them into the three types of dengue suggested by WHO. The authors used self-organizing maps and RF with clinical and laboratory data to identify characteristics that could be used as risk criteria for dengue severity. The results of this work are interesting because they show the characteristics of each disease group; however, it only focused on the diagnosis or classification of the disease and the recommendation of the best treatment option was not addressed.

Park et al. [43] implemented predictive models to classify patients with dengue. The authors used clinical and laboratory variables that fed into structural equation models. This was the first work that implemented structural equations applied to clinical data to develop predictive models; however, their work only used children and a small sample size to develop the models. It is difficult to generalize these results to patients of all ages. Additionally, the work was only focused on diagnosis and did not take into account the treatment of the disease.

The approaches and models previously developed and reported in the literature only meet two criteria corresponding to the use of ML techniques to generate the models, and the intuitiveness, extensibility and adaptability to increase their capabilities. It is important to remember that the clinical management of dengue involves not only diagnosis, but also treatment. The prediction or classification of dengue is insufficient if it does not support decision-making regarding treatment.

With this problem in mind, we proposed an ACODAT for the clinical management of dengue. The proposed approach considers data processing, classification and diagnosis of the patient into one of the three categories recommended by the WHO. Besides, our approach also provides an additional feature, which consists of the recommendation of the best treatment option (within a range of initially defined prescriptions) for a patient according to the type of dengue presented. The integration of different tasks that use AI techniques in the ACODAT was effective and allowed a more efficient clinical management of dengue patients, knowing that time is a critical factor for this type of patient. The proposed ACODAT was evaluated in different types of dengue. The results shown in previous sections demonstrate the diagnostic and prescriptive capability of the proposed approach.

In summary, our model is the only one that meets the four criteria defined in Table 10. Our approach uses AI techniques, not only for the classification of dengue, but also for prescribing the best treatment options (criteria A and B). To the best of our knowledge, there are no reports of automated systems for classifying dengue and recommending treatment (criteria C). Using only the variables used in conventional dengue diagnosis, our system can classify the clinical picture and recommend automatically treatment options. According to criteria D, our system is intuitive and easy to use, because the clinician only must enter age, signs, symptoms and laboratory tests. With this information, the system will automatically classify the patient and then recommend the best treatment options for that particular patient. Finally, our system is flexible and easily adaptable because it is possible to add new tasks to the cycle to consider other important aspects of dengue.

## 7 Conclusions

This paper proposed a clinical DSS for dengue using ACODAT. The objective was to develop a system that allows the processing of data, classification of the patient according to the type of dengue, and based on this last characteristic, recommendation of the best treatment option from a list of available treatments. The ACODAT developed has the ability to prepare the data and process them so that they are ready for the next task of the cycle. The AI techniques used, ANN and SVM, have the ability to correctly classify patients with high performance. The GA used in the last task of the cycle has the potential to recommend (prescribe) the best treatment option according to symptoms, signs and laboratory tests. The joint use of data analysis tasks in a cycle had key advantages over separate approaches. One of them is time to diagnose. With the proposed approach, it is possible to diagnose and recommend automatically patient treatment. This is very important because the time to diagnose and treat dengue is crucial to avoid complications and death of patients. To the best of our knowledge, this is the first work that uses an autonomic approach to support the clinical management of dengue. In addition, it is the first work to propose a prescriptive model for the clinical management of this disease.

This study has several limitations. First, some variables involved in the overall assessment process by the medical professional were not available to be included in the implementation of the models. Second, the unavailability of cohort datasets (before/after) to verify whether the recommended treatment had a positive impact on patients' health. For this latter, it is necessary to validate the results of this study in real hospital environments.

Future work should be aimed at improving the models implemented using routine laboratory tests such as white blood cell counts, blood levels of liver enzymes and cytokines. In addition, the inclusion of comorbidities such as diabetes and arterial hypertension could improve the performance of the models due to the influence of these diseases on the severity of dengue. Finally, the creation of available datasets with prescriptive or treatment variables would be useful to validate the results of prescriptive models.

## Declarations

### Author contribution statement

William Hoyos: Conceived and designed the experiments; Performed the experiments; Analyzed and interpreted the data; Contributed reagents, materials, analysis tools or data; Wrote the paper.

Jose Aguilar and Mauricio Toro: Conceived and designed the experiments; Analyzed and interpreted the data; Contributed reagents, materials, analysis tools or data; Wrote the paper.

### Funding statement

This study was partially funded by Colombian 10.13039/100007637Administrative Department of Science, Technology and Innovation - COLCIENCIAS (grant number 111572553478).

### Data availability statement

Data will be made available on request.

### Declaration of interests statement

The authors declare no conflict of interest.

### Additional information

No additional information is available for this paper.
